# Paddy insect identification using deep features with lion optimization algorithm

**DOI:** 10.1016/j.heliyon.2024.e32400

**Published:** 2024-06-05

**Authors:** M.A. Elmagzoub, Wahidur Rahman, Kaniz Roksana, Md. Tarequl Islam, A.H.M. Saifullah Sadi, Mohammad Motiur Rahman, Adel Rajab, Khairan Rajab, Asadullah Shaikh

**Affiliations:** a Department of Network and Communication Engineering, College of Computer Science and Information Systems, Najran University, Najran, 61441, Saudi Arabia; b Department of Computer Science and Engineering, Uttara University, Uttara, Dhaka, 1206, Bangladesh; c Department of Computer Science and Engineering, Khwaja Yunus Ali University, Sirajganj, 6751, Bangladesh; d Department of Computer Science and Engineering, Mawlana Bhashani Science and Technology, Tangail, 1902, Bangladesh; e Department of Computer Science, College of Computer Science and Information Systems, Najran University, Najran, 61441, Saudi Arabia; f Department of Information Systems, College of Computer Science and Information Systems, Najran University, Najran, 61441, Saudi Arabia

**Keywords:** Convolutional neural network, Lion optimization algorithm, Principal component analysis, Linear discriminant analysis, Deep learning, Machine learning, Pest identification

## Abstract

Pests are a significant challenge in paddy cultivation, resulting in a global loss of approximately 20 % of rice yield. Early detection of paddy insects can help to save these potential losses. Several ways have been suggested for identifying and categorizing insects in paddy fields, employing a range of advanced, noninvasive, and portable technologies. However, none of these systems have successfully incorporated feature optimization techniques with Deep Learning and Machine Learning. Hence, the current research provided a framework utilizing these techniques to detect and categorize images of paddy insects promptly. Initially, the suggested research will gather the image dataset and categorize it into two groups: one without paddy insects and the other with paddy insects. Furthermore, various pre-processing techniques, such as augmentation and image filtering, will be applied to enhance the quality of the dataset and eliminate any unwanted noise. To determine and analyze the deep characteristics of an image, the suggested architecture will incorporate 5 pre-trained Convolutional Neural Network models. Following that, feature selection techniques, including Principal Component Analysis (PCA), Recursive Feature Elimination (RFE), Linear Discriminant Analysis (LDA), and an optimization algorithm called Lion Optimization, were utilized in order to further reduce the redundant number of features that were collected for the study. Subsequently, the process of identifying the paddy insects will be carried out by employing 7 ML algorithms. Finally, a set of experimental data analysis has been conducted to achieve the objectives, and the proposed approach demonstrates that the extracted feature vectors of ResNet50 with Logistic Regression and PCA have achieved the highest accuracy, precisely 99.28 %. However, the present idea will significantly impact how paddy insects are diagnosed in the field.

## 1 Introduction

The human population in the world increased at a sluggish rate up until the year 1700 because of the exceptionally high infant mortality rate. But the global population hit 7.888 billion in 2021. Enhanced healthcare delivery is mainly responsible for the rapid increase in population seen in recent decades. The UN (United Nations) estimates that by 2050, the global population will have reached 9.71 billion. By 2100, it will have reached 10.35 billion, driving up demand for agricultural products and prompting a corresponding increase in food production. To satisfy the growing demand for food, biofuels, and other animal products brought on by an expanding population, crop yield production will have to rise by an average of two by the year 2050. To accomplish this objective, the yields of important crops will need to increase by 2.4 % yearly when they are only improving by approximately 1.3 % yearly. Rice is the 3rd most crucial cereal crop after wheat and maize in food production worldwide. The Food and Agriculture Organization (FAO) estimates that the annual rice crop yield in the developed area of 166 Mha is 745.17 mt, with a usual profitability of roughly five t/ha. As Lampe proposed in 1995, it is estimated that by 2025, 880 mt of paddy should be delivered, an increase of roughly 70 % to meet the rising population requirement [[1], [2], [3], [4]].

Rice is the most important crop grown for human consumption on earth since it provides more people with their daily intake of natural food than any other crop. Rice is responsible for providing 15 percent of the world's population with protein and 21 percent of the worldwide population with energy per capita, according to the International Rice Research Institute (IRRI). Although it has a relatively low protein content compared to other cereals, rice ranks highly in national quality [5]. However, Asia is the biggest supplier and buyer of rice, so its importance must be considered in other parts of the world. It is an increasingly economically essential agricultural crop in numerous emerging nations due to food diversity and immigration, and it is also becoming an essential agricultural grain in several developed industrialized countries, especially in the North American continent and the European Union. Additionally, many of the world's inhabitants rely on rice from paddy farms as their primary food source. Rice cultivation procedures conserve water, and using fertilizers can help to minimize methane emission levels while preserving the lives of millions of small-scale rice farmers [6,7].

Various infectious diseases and insects or pests continuously jeopardize the production of crops. Plant diseases and insect attacks are responsible for a loss of 20–40 % of the world's annual agricultural production, projected to cost the global economy $220 billion and $70 billion annually. Additionally, the agricultural output is reduced by between 8 and 10 percent annually as a result of several different diseases as well as the attacks of harmful insects. Remarkably, between 1950 and 2000, there was a more considerable proliferation of crop pathogens and pests in North America, in contrast to other parts of the world [8]. According to the agricultural community, approximately 37 percent of the annual rice harvest is lost to insects and disease states. Losses can be drastically reduced if a proper diagnosis is made quickly and accurately in conjunction with careful crop management. Several common insects, including the leaf folder, whorl maggot, and armyworm, are capable of causing rice crops to exhibit undeniable signs of damage during the early growth phases of the crop. Plants eaten by bugs have had their nutrients depleted, preventing them from being harvested. As a result of the insect's existence, the amount of food produced by the crops decreased. Farmers have been turning to pesticides and other chemical treatments for a long time to keep insects and other pests at bay. Increases in the use of pesticides for crop protection have been linked to human health problems and soil and water contamination. Conversely, this also raises the possibility that pests will evolve resistance to pesticides [1,9]. To exterminate the insect wreaking havoc on the rice crop, a qualified specialist needs first to determine the type of pests in accordance with the technical criteria in place.

Numerous innovative approaches have been developed to improve disease and insect detection, increasing crop yields and improving crop quality for the benefit of farmers and the people who work in agriculture. The fields of agriculture could benefit greatly from the application of Machine Learning (ML), Artificial Intelligence (AI), and Deep Learning (DL) techniques, which have the potential to provide vital information such as soil quality, ideal timing for sowing and spraying, and the areas most likely to get infested with pests. Farmers all throughout the world have been using these strategies to help them keep an eye on their crops [[10], [11], [12]]. A significant number of AI, ML, and DL-based solutions have been developed to supply farmers with data-driven decision-making help systems. This will aid farmers in protecting and increasing crop quality and output while decreasing the pesticides they need. To find paddy insects and diseases, many DL-based methods have been developed. With a 96 % success rate, the research [13] employed the radial basis neural network to categorize insect images based on their shapes and colors retrieved from the dataset. Also, the work [14] suggested a quicker R-CNN architecture comprising automated recognition algorithms for two different kinds of insects, which had an accuracy percentage of 90.7. In addition, several strategies were also developed with the use of ML and DL algorithms in order to identify paddy insects and diseases. The authors [15] utilized a mix of ML and Convolutional Neural Networks (CNN) to detect five different kinds of corn pests in the northeastern cold area. In the publication [16], researchers began reorganizing a massive dataset for identifying rice pests using Web crawler technology and manual inspection. Firstly, the study takes a transfer learning strategy, importing the weights of three deep models (ResNet50, VGG16, and MobileNet) that have been trained on ImageNet and applying them to the problem of identifying rice pests. The VGG16 classification model enhanced with transfer learning had the highest accuracy (84.39 %) and the most significant performance across all evaluation measures.

As one of the world's most important crops, rice is the subject of the work's survey [5], which focuses on the importance of precision agriculture in the industry's pursuit of growth. This study provides an overview and critical analysis of a number of papers published over the past eight years that focus on different approaches to identifying crop diseases, monitoring seedling health, and assessing grain quality. Yolov5, a deep learning model, is used in the study [17] to develop a simple and optimal model for detecting diseases in rice leaves. With the best precision, recall, and mAP values of 1.00, 0.94, and 0.62, respectively, the model has obtained a significantly superior identification rate. An approach based on fully convolutional networks (FCNs) was suggested in the article [18], and ten rice pests were chosen for experimentation. This work introduces a novel encoder-decoder to the FCN and a network architecture consisting of several sub-networks linked together via jump paths that utilize long leaps and shortcut links to ensure precise and subtle identification of insect boundaries. In addition, the network incorporates a conditional random field (CRF) module for insect-contoured enhancement and boundary localization. Lastly, a novel DenseNet system that introduces an attention mechanism (ECA) is suggested to concentrate on extracting insect edge features for efficient rice pest categorization. The presented model's overall recognition accuracy was achieved at 98.28 %. The research [19] proposes an ensemble-based model that uses transfer learning. This study investigates the potential of an ensemble model using a collection of previously trained models (Inception v3, Xception, VGG19, VGG16, and ResNet50). Approximately 82.5 percent accuracy can be achieved using the proposed ensemble model. The authors [20] suggested a convolutional neural network model that used transfer learning to categorize various pests. Across 40 different insect categories, the model had an average accuracy of 96.75 percent. The publication [21] also describes the development of a CNN algorithm using image processing to identify rice pests and diseases. This model achieved 90.9 percent accuracy, and the final results are kept in a documentation file for use in developing an Android app to detect rice pests and diseases.

Furthermore, a practical model based on a Convolutional Neural Network thoroughly studies and effectively categorizes the ten categories of agricultural pests in the paper [22]. GoogleNet achieved a maximum accuracy score of 94.61 % among all CNN models (ResNet50, ResNet152, VGG-16, and VGG-19). The authors [23] developed a deep learning-based model for rapidly classifying rice pests and paddy diseases. The caffeNet model, based on deep learning, provided an average accuracy score of 87 %. Additionally, the study [24] used deep residual learning to effectively classify agricultural pests against a complex background model. With three different approaches (AlexNet, ResNet-50, and ResNet-101), the proposed model could detect ten different agricultural pests against a complex background with a recognition accuracy of 89.33 percent. In order to identify and locate crop pests and rice diseases, the authors of the research [25] primarily contributed two architectures, VGG16, and Inception V3. The VGG16 model provided the most accurate results, scoring 97.12 % in detecting and categorizing rice diseases and crop pests. The study [26] constructed an effective model employing deep transfer learning. This model provided the best accuracy (89.33 %) using the AlexNet framework. Additionally, a novel CNN-based model for the early detection of ten prevalent rice illnesses was proposed in this article [27]. The suggested CNN model solves the multi-classification issue with the help of a regression learning technique, and it has an average accuracy of 95.48 percent. The research [28] developed a convolutional neural network-based model for insect recognition, which addresses the difficulties associated with the multi-classification of crop insects. To visualize the feature maps, researchers experimented with several distinct feature extraction networks, including ZF Net, VGG16, and VGG19, ultimately settling on VGG19 due to its superior accuracy of 89.22 %.

The objective of the paper [29] is to present a survey on various topics associated with the application of biometric authentication in healthcare monitoring systems. The current authentication is based on acquiring critical ECG signals through specified wearable devices compatible with 5G technology. To explore potential aspects that influence the signal-collecting procedure, the proposed review focuses on the characteristics of ECG signals and the present state of research concerning ECG authenticity. Furthermore, the survey discusses the psycho-physiological elements that come into play when using ECG data as a biometric feature in biometric identification systems. The article [30] proposed a new automated technique to diagnose kidney stones from CT scans reliably. Using deep learning and metaheuristics, the suggested method offered an innovative strategy. The primary goal was to develop a deep belief network (DBN) model that could reliably recognize kidney CT images by utilizing an upgraded version of a metaheuristic technique called the fractional order coronavirus herd immunity optimizer. The suggested model achieves a 97.98 % accuracy rate in simulations, which is higher than the other methods. A total of six market economies' fiat currencies are analyzed in the study [30], which are three developed (the euro, the pound sterling, and the yen) and three emerging (the yuan, the rupee, and the ruble) economies. Methods used in empirical research include Markov regime-switching regression (MRSR) analysis and symmetric, asymmetric, and non-linear causality testing. Regarding price and other return components, the results reveal that Bitcoin is causally related to the Chinese yuan and the Indian rupee. By showing that contact exists in contractionary regimes, the MRSR study supports these results. The value of Bitcoin is positively and significantly impacted by the appreciation of the Chinese yuan and Indian rupee during market downturns (like COVID), which may be attributable to market timing. After modifying three models to produce multiple MDSets (minimum dominating set) with minimum intersections for PPI networks, the authors of the preceding paper [31] used the minimization of metabolic adjustment (MOMA) algorithm to create a new framework called maximization of interaction adjustment (MOIA). With only a few iterations, the suggested algorithm can categorize all nodes in a PPI network as crucial, intermittent, or redundant using MOIA models.

To divide patient data into two categories—H1N1 influenza and COVID-19—paper [32] suggests a framework utilizing ML algorithms. The results of the experiments indicate that the Bayes network achieves an accuracy of 86.57 %, the naive Bayes classifier achieves an accuracy of 82.34 %, the multilayer perception algorithm achieves an accuracy of 99.31 %, the locally-weighted learning algorithm achieves an accuracy of 88.89 %, and random forest achieves an accuracy of 83.16 %. An innovative approach to skin cancer detection using deep learning and metaheuristics is detailed in the article [33]. The approach integrates a novel, enhanced metaheuristic approach based on the Multi-agent Fuzzy Buzzard Algorithm (MAFBUZO) with a multi-level optimal thresholding segmentation strategy that considers the input images' histograms. The study's findings demonstrate that compared to other methods, the suggested one yields superior results with NPV, PPV, Accuracy, and Specificity values of 0.95, 0.88, 0.94, and 0.93, respectively. For better identification of anterior cruciate ligament (ACL) injuries, the study [34] suggests a new hierarchical method. The procedure begins with applying pre-processing techniques to enhance the quality of the images. Next, the Co-occurrence Matrix (GLCM) and Discrete Cosine Transform (DCT) are utilized in conjunction with one another, and eventually, features from the images are extracted. Subsequently, the characteristics are inputted into a DBN that has completed classification training and is optimized via the “Improved Honey Badger Algorithm,” a novel metaheuristic technique. All previous methods have been outperformed by the proposed method, which has achieved the highest efficiency with 96 % accuracy, 98 % sensitivity, and 80 % specificity. The article [35] presents a novel approach to oral cancer diagnostics that uses deep learning techniques grounded in a metaheuristic approach. The study begins with three pre-processing techniques—gamma correction, noise reduction, and data augmentation—to improve the raw images' quality and quantity. ISSA, an improved version of the squirrel search algorithm, is then used to carry out the optimal selection of the network weights to achieve higher accuracy. As a reliable COVID-19-positive case detector, Deep Convolutional Neural Networks (DCNN) have traditionally been employed in the proposed study [36]. Additionally, the Chimp Optimization Algorithm (ChOA) is offered to construct a fast COVID-19 detector capable of parallel implementation. An accuracy of 99.11 % has been determined to validate the mixed ensemble model DCNN-ChOA.

To forecast profits in financial accounting information systems, the study [37] suggests a model that combines the deep gated recurrent unit (DGRU) with the improved marine predator algorithm (IMPA). Moreover, 5 DGRU-based frameworks are presented, and between these frameworks, the most accurate framework for profit forecasting is DGRU-IMPA. A new method for distinguishing between the five most prevalent kinds of pulse repetition interval modulation (PRIM) is presented in this paper [38], and it consists of four steps. The initial step is feature extraction using a DCNN. During the second phase, extreme learning machines, also known as ELMs, are utilized for prim pattern identification in real-time. Stage three of the research involves adjusting the interaction weights and biases using the biogeography-based optimizer (BBO), which increases the network's resilience. To address the growing complexity of the model, the current study presents an optimized variable-length internet protocol-based BBO (VBBO) in the final stage of the process. The model outperforms competing ELM-based benchmark models with an absolute accuracy of 97.05 percent. The research [39] was carried out to present a method that makes it possible to enhance the forecast of urban gas consumption by considering weather variables such as temperature, pressure, humidity, wind speed, and gas price. This study developed hybrid models by combining the categorical boosting (CatBoost) method with several meta-heuristic algorithms. Phasor particle swarm optimization, artificial bee colony, battle royale optimizer, grey wolf optimizer, Satin Bowerbird method, and fruit fly optimization method are all meta-heuristic algorithms used in the work. The outcomes demonstrated that out of all the hybrid models that were presented, the Catboost-PPSO model performed the best. Using the ZFNet network trained on breast mammography pictures, a novel approach to detecting unusual breast cancer was suggested in the paper [40]. To test the efficiency of the pre-processing step, the Wiener and CALHE filters are utilized at the beginning of the procedure. An extreme learning machine (ELM) replaced the last few layers. A technique for determining the optimal amount of structural layers to replace was also detailed. A better categorization performance was the ultimate motivation for developing ELM. An enhanced version of the Chimp Optimization Algorithm, known as SWChOA, was used to achieve this. According to the outcomes of experiments that utilized a 10-hold-out validation, the method ZFNet-SWChOA-ELM has the highest level of performance.

An algorithm that takes its cues from the natural world, the ChOA mimics how chimpanzees think and hunt. To complete a hunt in this algorithm, one must drive, block, pursue, and attack. Like other iterative algorithms, ChOA's sluggish and premature convergence results from the simplest possible modeling of the hunting process, which is driven by the novelty of the method. In order to address these shortcomings, a study [41] presents six spiral functions and two new hybrid spiral functions (SEB-ChOA). They compare SEB-ChOAs to three sets of optimization algorithms: the most popular ones, such as Genetic Algorithm (GA) and Particle Swarm Optimization (PSO), as well as less well-known ones, like Slime Mould Algorithm (SMA), Marine Predators Algorithm (MPA), Ant Lion Optimization (ALO), and Henry Gas Solubility Optimization (HGSO). The authors also look at two sets of practically new optimization algorithms, jDE100 and DISHchain1e+12, which won the 2019 IEEE CEC06 competition, and two sets of secondary optimization algorithms, EBOwithCMAR and CIPDE, as better options. Compared to the top optimizers, jDE100 and DISHchain1e+12, the SEB-ChOAs achieved first place in nearly all benchmarks and showed highly competitive results. Based on the statistical data, the SEB-ChOA optimizer is superior to the following: PSO, GA, SMA, MPA, ALO, and HGSO. It also achieves results that are on par with jDE100 and DISHchain1e+12.

The research [42] suggests using a DCNN to create a reliable active sonar image classifier. The LeNet-5 is the simplest deep network with the fewest parameters to achieve a low-complexity real-time classifier. An extreme learning machine (ELM) takes the role of the three fully connected layers to allow for a training and testing phase that happens in real-time. The input layer parameters of the ELM are notoriously difficult to adjust, so the grey wolf optimizer (GWO) is employed in this study to do just that. The authors model the sonar problem as a multimodal function, which is different from prior research works, and consider the problem's features. Competent researchers evaluate the outcomes using traditional DCNN, Block-wise Classifier (BWC), and Matched Subspace classifier with Adaptive Dictionaries (MSAD). Based on the inquiry results, the created model outperforms all other benchmark models in terms of accuracy, with an average of 98.69 %. Ensuring precision and reliability in engineering structure design and operation relies heavily on distance measurement. The study [43] suggests a framework that uses resilient and cost-effective ultra-wideband radio technology to wirelessly sense distance. It also introduces an ML approach that increases measurement accuracy by including error mitigation techniques and is based on an extreme gradient boosting decision tree. The results showed that the distances were accurately measured within a millimeter. The suggested approaches show a tradeoff between precision and frequency of distance measurement, exhibiting the required accuracy, cost-effectiveness, and environmental robustness. To attain both high frequency and high accuracy simultaneously for distance measurement, the research [43] provides two machine learning methods. The first method includes a convolutional neural network, a module for lengthy short-term memory, and a regression module. In the second method, two random forest models are combined. Implementing these two methods into measurements taken by ultra-wideband (UWB) sensors mounted on a highway bridge resulted in higher measurement accuracy and output frequency than the state-of-the-art methods.

Technological advancements have helped farmers increase their crop yields while keeping costs down. The complexity of the problem meant that more than the developed methods were needed for detecting paddy insects and diseases. Additionally, the use of deep learning techniques in rice pest detection is hindered by the fact that some existing open-source datasets have either insufficient sample sizes or suffer from inter-class and intra-class volatility and data imbalance difficulties. Therefore, there should be a need for more precise and individualized strategies. That's why the proposed study's main aim is to use an autonomous system for the paddy field that uses cutting-edge techniques like ML, DL, and optimization algorithms to identify and classify pests. The following representations highlight the contributions made by the suggested article.

⁃ The current research presents a method for detecting pests in rice fields through automated inspection tools such as DL, ML, and a feature optimization algorithm.
⁃ The suggested article utilized seven classic ML classifiers to identify and classify paddy insects.
⁃ The features of a single image of the paddy are extracted and analyzed by employing only the feature extraction portion of the five pre-trained CNN architectures (including VGG16, VGG19, Inception V3, ResNet50, and Xception).
⁃ In the feature optimization technique, the Lion Optimization Algorithm (LOA) is employed in the present work to find the best features among the selected attributes using feature selector techniques.
⁃ In order to determine the most critical attributes from the data retrieved by the pre-trained architectures, the present framework employs multiple feature selector approaches, such as PCA, RFE, and LDA.

The manuscript is divided into five sections. In Section 2, the proposed system's methodology is presented and studied in detail. The results of the suggested study as well as the potential solutions, are presented in Section 3. Discussion and comparison with the exiting work is presented in Section 4. Lastly, the proposed research's final findings and future goals are presented in Section 5.

## 2 Materials and method

**Fig. 1:**
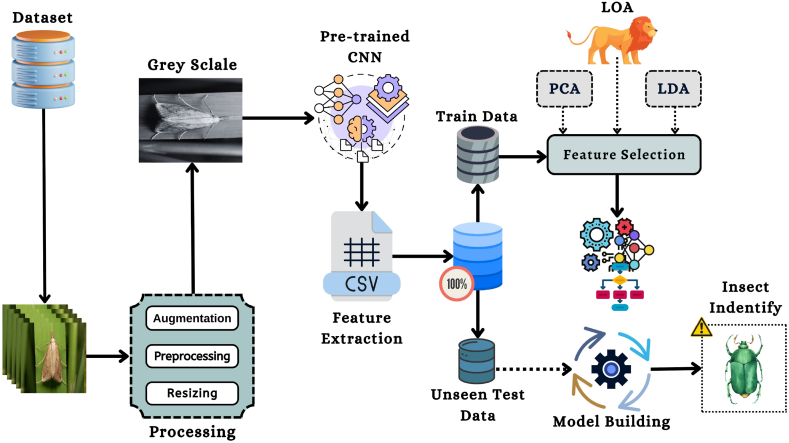
The overall methodology of the proposed framework.

Here, the methodology of the proposed research is described. This section represents four subsections connected to one another; these are gathering the dataset of the proposed study, feature extract with the help of DL techniques of pre-trained CNN algorithms, utilizing feature selector algorithms and optimization methods with the standard ML techniques. The suggested model's overall architecture is illustrated in Fig. 1, and in the initial step, the dataset of the paddy insects will be collected from the secondary source. In the second step, many image pre-processing techniques are applied to the images of the dataset. For the essential feature extraction from the dataset images, the proposed model will send the pre-processed images to the pre-trained CNN models in the third step. Fourthly, many feature selector algorithms have been utilized in the suggested approach to find more accurate features from the image features of the pre-trained models. The last step is splitting the dataset into testing and training for the model assessment. As for the training data, which is fed to the standard ML algorithms to train the current model, the testing data is used to measure the proposed model's performance. After finding the exploratory results of the present model, a comparison between the preceding works has been described.

### 2.1 Dataset preparation

The collection of samples that have the same attributes is called a dataset. Data should be fed into the present approach to train the ML classifiers. After that, model validation/testing data is given to measure the model's performance. From Kaggle (an open data source), the dataset of the proposed work was collected [18]. The collected dataset has two data categories: paddy with pest (yes type) and paddy without pest (no type). There are 135 images in the first category and 513 in the second category. Samples of the dataset images are shown in Table 1. The corresponding dataset splitting for training and testing of the present model is shown in Table 2.

**Table 1:** Sample of each class of the dataset.

Type	Paddy With Pest	Paddy Without Pest
*Paddy Insects*

**Table 2:** Real dataset with splitting for testing and training.

Insect Type	Class Type	Training Images	Testing Images
Paddy Insect	Paddy With Pest	135	26
	Paddy Without Pest	413	82

### 2.2 Pre-processing

This section will discuss the methods of image pre-processing. Through image pre-processing, the present model can accurately identify the paddy insects as the dataset contains noise or other unnecessary things, which will cause the model not to be more accurate. Image filtering and image augmentation techniques are used in the proposed study, where the augmentation techniques are used to fix the small dataset problem. In contrast, the image filtering strategy eliminates the dataset's distractions.

#### 2.2.1 Image augmentation

A method for artificially expanding the size of a dataset is image augmentation. This procedure yields fruitful results despite the limitation of working with a limited dataset. Horizontal flip, rotation, vertical flip, shear, 20 % contrast increase, reflection, and highlighting brightness are all standard image enhancement procedures performed for image augmentation. In order to generate a sizeable dataset for use in insect identification in rice fields, the proposed research has successively carried out this procedure. After applying picture enhancement strategies, the size of the present dataset is shown in Table 3.

**Table 3:** Total dataset with splitting for testing and training after.

Insect Type	Class Type	Training Images	Testing Images
Paddy Insect	Augmented Paddy With Pest	505	100
	Augmented Paddy Without Pest	513	104

#### 2.2.2 Image filtering

To eliminate the noise already present in the image, the technologies of image filtering have been used in the present work. Different filtering methods are used to filter the images, including:

**Gaussian Filter:** These filtering techniques are the most frequently used filter among the other linear filters. To blur the image or reduce the noise of the image this method is used. Eqs. (1), (2)) represents the Gaussian blur strategy,

(1) $$G\left(x\right)=\left(1/\surd \left({2\pi \sigma }^{2}\right)\right)\;e^{\left(-x2/\;2\;\sigma 2\right)}$$

(2) $$G\left(x\right)=\frac{1}{\sqrt{2\pi \sigma^{2}}}e^{-\frac{x^{2}}{2\sigma^{2}}}$$

**Average Filter:** Filtering based on the mean or average value of the window's pixels rather than the center point is an average filtering technique. It is helpful in a wide range of different situations. The window, sometimes called the kernel, is typically square but can be any size.

**Median Filter:** Regarding spatial filters, the median filter is another sliding-window variant of the average filter. Nevertheless, rather than the center point, it uses the median score of all of the window's pixels as its substitute.

### 2.3 Feature extraction

The suggested study's mechanism for extracting features is outlined here. The workflow for the extraction of features, together with its associated algorithmic explanation, will be presented first. The method for choosing features will be described after that. In addition, the algorithmic explanation and operational principles of the Lion Optimization Algorithm (LOA) will be described in this part. The subsection provides an explanation of feature vectors as well as the working operations of classic Machine Learning algorithms.

#### 2.3.1 Feature extraction working principle

**Fig. 2:**
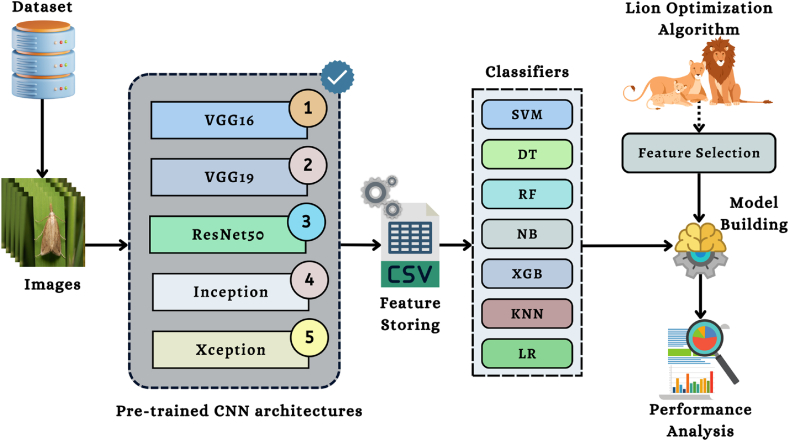
Pipeline of the feature extraction of an image.

The proposed method employs five different pre-trained CNN models to extract features from a single image. Fig. 2 depicts the operational process that is involved in the feature extraction. In the figure, images from the dataset are supplied to the pre-trained CNN models in order to extract image features. The image features have been extracted using five pre-trained models, including VGG16, VGG19, Xception, Inception V3, and ResNet50. After extracting the features for categorizing the images, 7 classic ML algorithms were constructed. In addition, the LOA is implemented as a strategy for feature selection in this investigation so that researchers can work with high-quality features. The present approach then assesses the significance of the results for each category. The entire pipeline uses Algo. 1 to extract features from an image.

Algo. 1 shows a comprehensive and efficient breakdown of how to extract features from a single image. The system initialized the dataset with the required number of insect images to kick off the process. P and Q stand for the original and adjusted images, respectively. The pseudocode serves as a loop structure for defining the feature vectors.

Algorithm 1 A working mechanism to extract feature vectors of the proposed work.

**Table:** 

*Input****:* 2D Images**					
Output**: Feature Vectors**					
Initialization	:				
	1.	a = 2A-1, Here, A = 1, 2,3, 4………….a			
	2.	P ← System input (Image)			
	3.	Q_a_ ← Using the kernel size a×a, apply the median filter to the input Image P.			
	4.	R_u_ ← Respective Output (Feature vectors)			
Start	:				
	1.	**for** each A:			
	2.		Find Q_a_		
	3.		Use (P, Q_a_) to get R_a_ │ R_a_ {V_0_, V_1_,. . . , V_14_}		
	4.		R_u_ ← R_a_		
	5.	**End for**			
	6.	Show R_u_			
End	:

#### 2.3.2 Description of the pre-trained algorithms

**Fig. 3:**
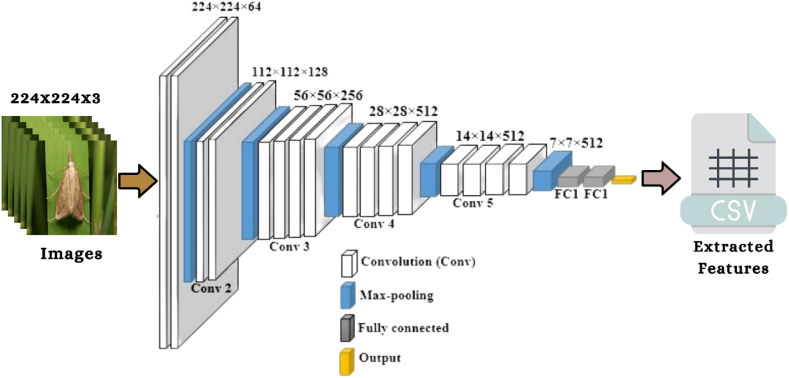
The architecture of VGG16 algorithm [50].

**Fig. 4:**
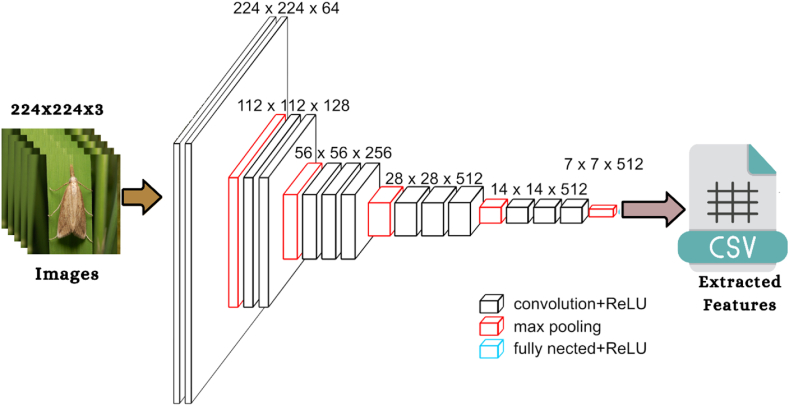
The framework of VGG19 algorithm [50].

The study utilized five pre-trained convolutional neural network models to extract the properties of each image. This research work provided the conceptual framework of the VGG16 and VGG19 algorithms to study the influence that the depth of a network has on the accuracy of forecasting. On the 2014 ImageNet Challenge, the two proposed models had the highest prediction accuracy. When increasing the number of weight layers from 16 to 19, the architecture utilized 3 × 3 convolutional filters of small size, contributing to the significant enhancement achieved. 13 of the VGG16 layers are involved with the convolutional operation. In contrast, five correspond with the pooling operation, and three are fully linked layers. On the other side, the design of VGG19 has 16 convolutional layers, five layers with pooling, and three fully connected layers. The VGG19 method requires an input image with a resolution of 224 × 224 × 3 and will use 4096 features (the result of the final layer of the feature extraction section) [44]. Fig. 3, Fig. 4 depict the corresponding framework of the VGG16 & VGG19.

**Fig. 5:**
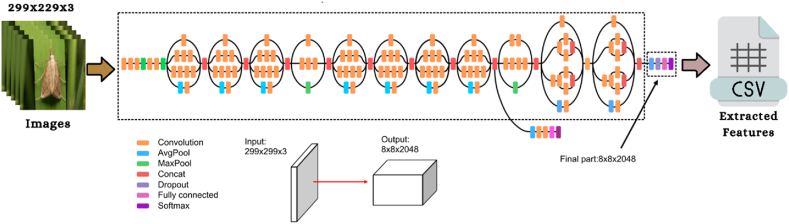
The architecture of InceptionV3 algorithm [50].

Additionally, the InceptionV3 framework will then be used to do feature extraction. InceptionV3 is also known as GoogleNet [45]. It is a pre-trained model of CNN algorithms. The Inception model consists of 22 layers and 5 M parameters; filter sizes of 1 × 1, 3 × 3, and 5 × 5 are utilized to extract features at various sizes. The network's efficiency is reduced since the 5 × 5 convolutional filters were swapped out for two 3 × 3 convolutional filters to reduce computation. The 48-layer. Finely-tuned structure of InceptionV3 helps to reduce overfitting. Extracting the hidden features of insect data is done with the use of the InceptionV3 model, as shown in Fig. 5.

**Fig. 6:**
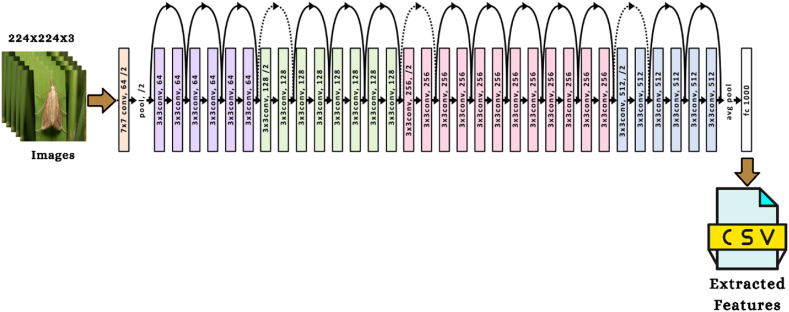
The framework of ResNet50 algorithm [50].

The suggested study also uses the ResNet50 CNN architecture that has already been pre-trained to extract features from insect images. The ResNet50 network has 50 layers and around 2 million parameters. The ResNet50 framework is made up of several individual parts. The first component consists of 64 kernels, each with a fully connected layer, a max-pooling layer, and a convolutional layer. The disappearing problem is fixed, and the enhancement layer reduces degradation. As input, the model will take an insect picture with dimensions of 224 × 224 × 3, generating 4096 features for each image (these are the results of the feature extraction section's final layer) [45]. Fig. 6 depicts the architecture of the ResNet50.

**Fig. 7:**
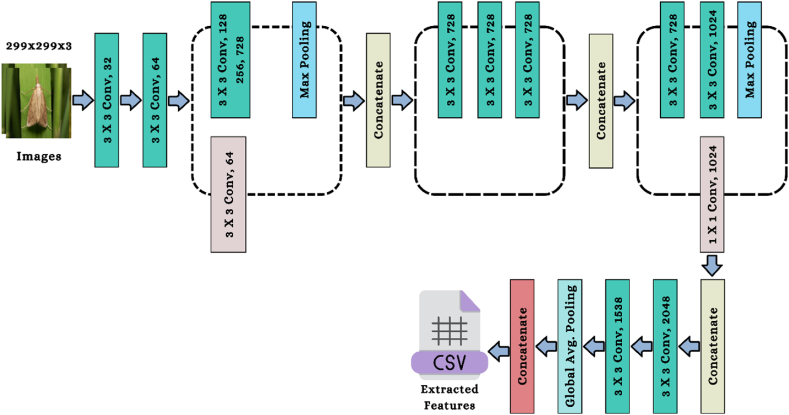
The architecture of the Xception algorithm.

The Xception is a pre-trained CNN technique used in this study to extract features from an image. The architecture of the Xception model is shown in Fig. 7. The Xception method's framework used 36 convolutional layers to make the model as effective as possible when it came to extracting features from data. Except for the initial and final modules, all 14 modules that comprise the 36 convolutional layers are surrounded by the linear residual connections. The Xception architecture comprises convolutional layers stacked linearly stacked and may be taught independently in great depth [46].

#### 2.3.3 Feature selection models

**Fig. 8:**
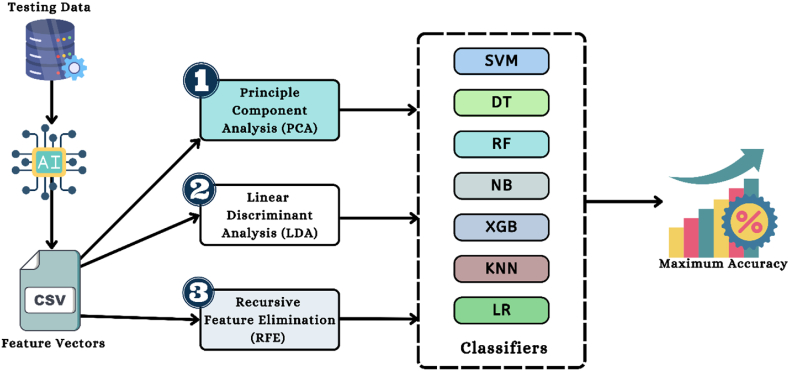
Feature selector model's framework of the proposed study.

This section explains how Fig. 8, the feature selection model for the proposed study, works. RFE, LDA, and PCA are three of the most outstanding feature selection methods used in this study's development. Feature reduction using PCA and LDA models is quite effective [47,48]. PCA is a method of unsupervised learning that tries to increase the amount of the dataset's individuality. On the contrary, LDA and RFE are examples of supervised learning models. These models focus on a feature vector domain in order to improve a group's ability to be discriminated from one another. The following describes certain mathematical expressions relating to LDA and PCA.

In PCA, the covariance matrix is constructed in a way that maximizes efficiency. For PCA to be constructed, the covariance matrix must be of symmetric d×d dimensions. Here d is the number of dimensions of a dataset that includes pairwise covariance between multiple critical characteristics. To illustrate, let $X_{j}$ and $X_{k}$ stand for two features of the intended audience. Covariance is calculated using the following Eq. (3).

(3) $$\sigma_{jk}=\frac{1}{n}\sum_{i=1}^{n}\;\left(x_{j}^{\left(i\right)}-\mu_{j}\right)\left(x_{k}^{\left(i\right)}-\mu_{k}\right)$$

On the other hand, the LDA model is effective since it contains of five interconnected phases. All of these operations are detailed in Algo. 2. Input features from images are used by this this method to generate optimal features.

Algorithm 2 Working procedure of LDA in feature selection [50].

**Table:** 

*Input****:* Image Features**				
Output**: Best *Features***				
Start	:			
	1.	Compute the d-dimensional mean vectors for all classes of the dataset.		
	2.	Calculate the scatter matrices, specifically the scatter matrix across classes and the scatter matrix within classes.		
	3.	Evaluate the eigenvectors of the scatter matrices' (e_1_, e_2_, e_3_…*.*e_d_) and corresponding eigenvalues (ƛ_1,_ ƛ_2,_ ƛ_3,_ ƛ_4……*.*_ ƛ_d_)*.*		
	4.	Arrange the eigenvectors by decreasing eigenvalues, and select the k eigenvectors with the greatest eigenvalues to create a matrix W of dimensions d × k. (Each column represents an eigenvector).		
	5.	The samples will be converted onto the new subspace using this d × k eigenvector matrix. This outcome can be summarized using matrix multiplication: Y= X × W (where X is an n × d-dimensional matrix representing n samples and y are transformed n × k-dimensional samples in the new subspace).		
End	:

RFE is the feature selection approach which is used in the proposed article. By recursively deleting each feature, RFE, a wrapper feature selection approach, may repeatedly assess the significance of features. RFE aims to maximize performance by reducing the number of features to a manageable size or to meet a predetermined number of features [49]. The working procedures of the RFE for selecting features are depicted in Algo. 3.

Algorithm 3 Working procedure of RFE in feature selection [50].

**Table:** 

*Input:* Images Features							
**Output:** Best Feature *Vectors*rowhead							
**Initialization**	:						
	1.	A= X-1, Here X = Number of features in a specific convolutional neural network (CNN) model.					
	2.	B ← The number of classes					
	3.	P_u_ ← The number of training data					
	4.	Q_u_ ← The number of testing data					
	5.	V_f_ ← Feature Vectors (Respective Best features)					
	6.	F_s_ ← Number of selected optimal feature vectors					
**Start**	:						
	1.	feature = *SVMFeatureSelection* (P_u,_ Q_u_)					
	2.	**If** (The number feature>0.5)		:			
	3.		F_s__=_ Selected features				
	4.		V_f__=_ Add no. *selected* features (all the F_s_)				
	6.	**End if**					
	5.	Store (the best features) V_f_					
**End**	:

### 2.4 Working procedures of proposed lion optimization algorithm (LOA)

Metaheuristic algorithms have gained a lot of interest from scholars and practitioners in recent years for their ability to solve complicated optimization issues. Thus, many metaheuristic algorithms have been developed over the past few years. Several natural occurrences have served as inspiration for these algorithms, such as Whale Optimization Algorithm (WOA) [51], Particle Swarm Optimization (PSO) [52], Salp Swarm Algorithm (SSA) [53], Chimp Optimization Algorithm (ChOA) [[54], [55], [56]], Dragonfly Algorithm (DA) [57], Lion Optimization Algorithm (LOA) etc. For the proposed framework, the LOA is used to find the best features from the extracted data of the feature selection process.

**Fig. 9:**
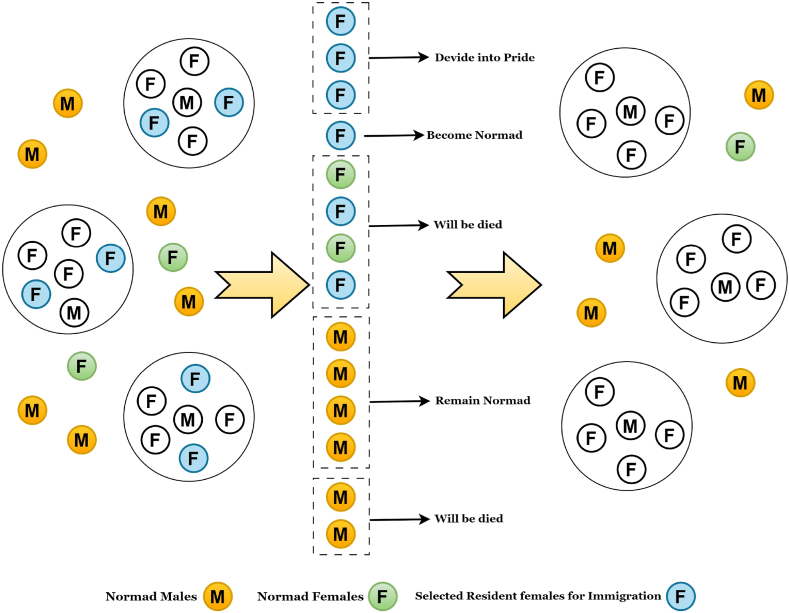
The process of lion population equilibrium in LOA classification with the classic ML classifiers.

**Fig. 10:**
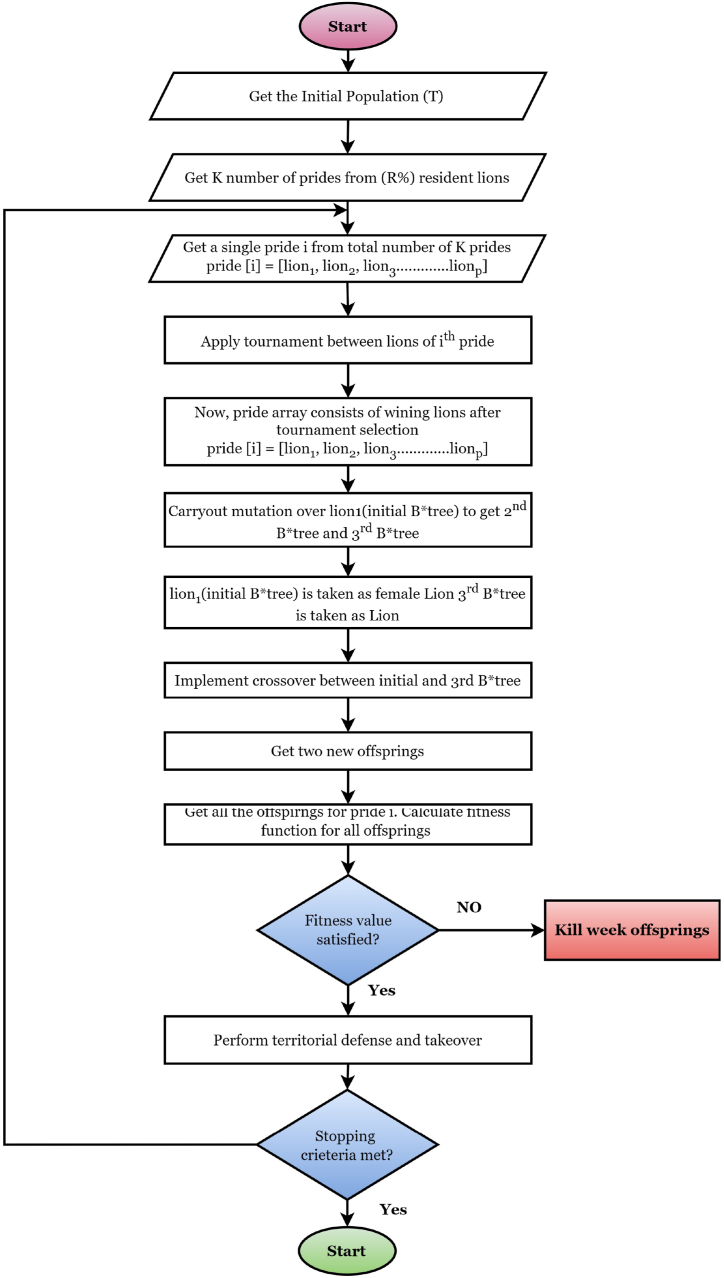
The working procedure of LOA in the proposed study.

The working mechanism of the LOA is described in this section. Lions are highly socially sensitive animals with traits of cooperation and aversion. The social organization of wild lion behavior is divided into two categories: residents and nomads. A lion tribe often comprises five females and their cubs of both sexes, as well as one or more adult males. When young male lions develop, they frequently rule out of their tribe. The lions then become nomads, living in pairs or alone. This process is continued like becoming the superior of a tribe or the normad and vice-versa. Male and female lions in the same pride cooperate to protect one another and their pups from other predators. Over territory, the entire pride of lions can engage in deadly conflicts. When they have places that overlap, they may dispute who gets to keep them. Such clashes are becoming increasingly common. When a new nomad male takes command of pride, the lion's social behavior begins. During this takeover, the male lion kills all existing young in order to procreate immediately. The mating and territorial takeover behavior of lions is utilized in this work to optimize an algorithm. Fig. 9 depicts the lion's social behavior. On the other hand, with the social behavior of the lion, we can interpret an algorithm using B*tree, which denotes a single or normad lion. The working principles of this algorithm are depicted in Fig. 10. In this figure. The algorithm is divided into four types of operations: (1) the generation of the initial population, which is identically responsible for structuring the B*tree through the process of mutation; (2) the process of mating, which leads to the determination of new B*trees through the process of mutation. (3) Territorial defense among the lions, and lastly (4) Territorial takeover for selecting the new best solution (B*tree) by eliminating weak fitness function [58,59]. LOA has many operations, which are broadly explained below:

**Initialization:** The initial stage of LOA is to produce the population in random order across the solution space. ''Lion'' is the name of every solution in this algorithm. A lion is depicted in Eq. (4) in an $N_{var}$ Dimensional optimization problem:

(4) $$Lion=\left[x_{1},x_{2},{\;x}_{3},\ldots ,x_{N_{var}}\right]$$

Evaluating the cost function yields the cost (fitness value) of each Lion, which is shown in Eq. (5):

(5) $$fitness\;value\;of\;lion=f\left(Lion\right)=f\left(x_{1},x_{2},{\;x}_{3},\ldots ,x_{N_{var}}\right)$$

In the first stage, a random number generator is used in the search space to create $N_{pop}$ solutions. Nomad lions are selected at random from among the generated solutions. The remaining individuals will be assigned to P prides at random. Throughout optimization, the algorithm maintained that each solution had a specific gender. This is why, in each pride, the remaining members are referred to as males and the remaining percentage as females, which ranges from 75 % to 90 % of the total population. The corresponding percentage for nomadic lions is %(1−S). During its search, each lion establishes its preferred spot. Each pride's region is defined by its marked positions. Hence, the area of each pride is determined by its members' indicated positions or the places they frequent the most.

**Hunting:** The hunting process begins with a group of females in each pride searching for prey to feed the pride. To successfully encircle and capture their prey, these hunters employ precise tactics. When it came to hunting, lions generally followed an approach that was roughly similar to one another. These lions are classified into three distinct stalking positions: left wing, center, and right wing. While hunting, every lioness adjusts her posture according to how it concerns the others in the pride.

The center of hunters is considered as the dummy prey (PREY), which is defined as Eq. (6):

(6) $$PREY=\sum hunters\frac{x_{1},x_{2},{\;x}_{3},\ldots ,x_{N_{var}}}{number\;of\;hunters}$$

The hunter's fitness improves to the point that the PREY escapes from him, and the new location of the PREY is determined by the following Eq. (7):

(7) $$PREY^{\prime }=PREY+rand\left(0,1\right)\times PI\times \left(PREY-Hunter\right)$$

The variables PREY, Hunter, and PI represent the current position of the prey, the new position of the hunter who attacks the prey, and the percentage of improvement in the hunter's fitness, respectively. In order to simulate the behavior of the hunter groups, the newly created hunter spots split evenly between the left and right wings, as shown in Eq. (8):

(8) $$Hunter^{\prime }=\left\{ \begin{array}{c} rand\left(\left(2\times PREY\;-\;Hunter\right),PREY\right),\left(2\times PREY\;-\;Hunter\right)<PREY\\ rand\left(PREY,\left(2\times PREY\;-\;Hunter\right)\right),\left(2\times PREY\;-\;Hunter\right)>PREY \end{array}\right.$$

while PREY denotes the present location of the prey, Hunter denotes the current location of the hunter, and ${\text{Hunter}}^{\prime }$ represents the new location of the hunter. Additionally, as the following Eq. (9), the new roles of Center hunters are created:

(9) $$Hunter^{\prime }=\left\{ \begin{array}{c} rand\left(Hunter,PREY\right),Hunter<PREY\\ rand\left(PREY,Hunter\right),Hunter>PREY \end{array}\right.$$

**Moving Toward Safe Place:** As previously stated, some of each pride's female members engage in hunting activities. Those surviving females make their way to one of the territory locations. When it comes to improving solutions in LOA, each pride's territory contains each member's personal best locations so far. This helps LOA save the best solutions achieved so far during the period of iteration, which is valuable and accurate information. As a result, a female lion's new position can be represented as Eq. (10):

$$Female\;Lion^{\prime }=Female\;Lion+2D\times rand\left(0,1\right)\left\{R1\right\}+U\left(-1,1\right)\times \tan\left(\theta \right)\times D\times \left\{R2\right\}$$

(10) {R1}⋅{R2}=0,∥{R2}∥=1

where FemaleLion is the lion's current location, and D is the distance from her current position to the place in the pride's territory that was chosen at random during the tournament. The vector {R1} starts at the spot where the female lion was before and points in the direction of the position that has been chosen. As a result, {R2} is orthogonal to {R1}.

Now, the strategy that will be used for the tournament section is explained. To begin, a lion has succeeded if it improved its best position at the last LOA iteration. Within group P, the lion i success at iteration t is defined as Eq. (11):

(11) $${S\left(i,t,P\right)=\{}_{0\;{Best}_{i,P}^{t}={Best}_{i,P}^{t-1}}^{1\;{Best}_{i,P}^{t}\;<\;{Best}_{i,P}^{t-1}}$$

where, ${\text{Best}}_{i,P}^{t}$ is the best position that lion i has found up until iteration t. Additionally, when the lions' success rate is high, it means they've settled on a location that isn't optimal. In the same manner, a low success rate indicates that the lions are aimlessly circling the best option without making any real progress. Because of this, it is a practical component for determining the element size of a tournament and the equation for compute $K_{j}\left(s\right)$ is given below in Eq. (12):

(12) $$K_{j}\left(s\right)=\sum_{i=1}^{n}S\left(i,t,P\right)\;j=1,2,..,P$$

The number of lions in pride j that improved their fitness in the last iteration is denoted by $K_{j}\left(s\right)$ and the success values are used to calculate it. Here, n is the total number of lions in the pride. Therefore, the number of tournaments in each pride changes with each cycle. This means that tournament sizes and variety go up as the success value drops. Hence, here is how the tournament size is determined as Eq. (13):

(13) $$T_{j}^{Size}=\max\left(2,celi\left(K_{j}\left(s\right)/2\right)\right)\;j=1,2,..,P$$

**Roaming:** For several reasons, any male lion in a pride is free to traverse the territory of that pride. The lion will visit a random selection of %R of the pride's area to mimic the actions of resident males. Keep his best-visited solution up-to-date while wandering if the resident man finds a better spot than its existing best position. In order to find a better solution, the LOA uses roaming, a robust local search. The lion's approach to the chosen region is x units away, where x is a uniformly distributed random number, and the distance from the male lion's current location to the selected territory region is represented by d. Eq. (14) is given below:

(14) x∼U(0,2×d)

**Mating:** Lions' existence depends on mating, allowing pride members to share knowledge. Every pride has at least one resident male lion and a matrilineal population of female lions. These males are chosen randomly from the same pride as the mother for mating purposes. One key difference is that nomadic lionesses only mate with one of the randomly chosen males. The mating operator uses a linear combination of parents to produce two new offspring. After selecting a mother lion and a male or males to breed with, the following Eq. (15) govern the birth of cubs:

(15) $${Offspring}_{j}\;1=\beta \times {Female\;Lion}_{j}+\sum \frac{1-\beta }{\sum_{i=1}^{NR}S_{i}\times {MaleLion}_{j}^{i}\times S_{i}}$$

$S_{i}$ is set to 1 if male i is chosen for mating; otherwise, the value will be 0, where j is a dimension. NR means the total number of males living in pride, and β is a number produced randomly from a normal distribution with a mean of 0.5 and a standard deviation of 0.1. In every generation, a male and a female progeny are chosen randomly. One of the resulting progeny has a mutation applied to each gene with a probability of (%Mu). Instead of using gene value, a random integer is used. When a mother LOA breeds with her cubs, the offspring get traits from both parents.

**Defense:** As they reach maturity, male lions in pride become combative and fight with other males. Males who have been beaten give up their dignity and become nomads and travellers. The resident male lion is forced to leave the pride and become a nomad if a nomad male lion is mighty enough to attempt to take over the pride by fighting its males. There are two primary phases to the LOA process for defense operators one is protection from newly-matured resident males, and the other one is protection against male nomads.

**Migration:** The natural phenomenon of a lion changing its lifestyle, such as moving from one pride to another or becoming a nomad, serves as an inspiration for this phenomenon. When a resident female lion migrates to a new pride, it brings the diversity of her former pride. But the lion's travel and switch lifestyle provide a link for communication. The population's S% governs the maximum number of females allowed in each pride. Some women were chosen at random to become nomads by the migration operator. The size of the migratory female population equals the sum of the surplus female population and %I of the maximum female population for each pride. As soon as a female is chosen to become a nomad, she is separated into two groups, new nomads and elderly nomads, based on how fit she is. After that, prides are populated with the finest females, chosen randomly to replace the migratory females. This method facilitates communication amongst prides and preserves the overall population's diversity.

**Lions' Population Equilibrium:** Since lion populations are inherently stable, the total number of lions will be managed after each cycle. Nomad lions with the lowest fitness values will be eliminated, taking into account the maximum allowed number of each gender.

**Convergence:** The convergence of an optimization algorithm's iterative results is based on the stopping condition, which is often the CPU time, maximum iterations, number of iterations without improvement, etc [58,59].

### 2.5 Classifiers

Traditional ML classifiers were used in the proposed work to give a prediction of insect detection in rice fields. 7 classic algorithms were used to do the deception task. The algorithms are namely, XGB (Extreme Gradient Boosting), LR (Logistic Regression), DT (Decision Tree), KNN (K-Nearest Neighbor), SVM (Support Vector Machine), NB (Naïve Bayes), and RF (Random Forest) [60].

**Extreme Gradient Boosting (XGB):** The practical ML approach XGB can help with both analysis and decision-making. More specifically, XGB is an algorithm that employs gradient-enhanced decision trees. It has been utilized by a large number of researchers from all around the world in attempts to fine-tune the ML models.

**Decision Tree (DT):** The decision tree is a supervised learning approach that utilizes a tree-based data structure to perform the result. The DT provides a visual representation of criteria for making a decision based on the characteristics of a data set. The tree's outer branches represent rules, while features of the dataset are mirrored in the nodes closer to the tree's root. The DT also provides a quick and easy method for displaying Boolean values in graphical form. The following Eq. (16) provides the solution for DT:

(16) $$Information\;Gain,S=S=-P\left(\frac{1}{true}\right)\log_{2}\;P\left(\frac{1}{true}\right)-P\left(\frac{0}{false}\right)\log_{2}\;P\left(\frac{0}{false}\right)$$

**Logistic Regression (LR):** LR is a technique of supervised classification that is used to compute the possible values of the target variables. Due to the binary nature of the target variable, this model only allows for two possible outcomes (1/yes or 0/no). There are three distinct kinds of LR: binary or binomial, multivariate, and ordinal. In order to determine LR, the following Eq. (17) is utilized.

(17) $$y=b_{0}+b_{1}x_{1}+b_{2}x_{2}+b_{3}x_{3}+\ldots +b_{n}x_{n}$$

Support **Vector Machine (SVM)**: SVM can be used as either a regression or classification method, depending on the amount of information available. To facilitate the incorporation of new information, SVM helps to partition n-dimensional spaces into discrete classes. The kernel trick approach of SVM was used to perform the classification, and the corresponding kernel trick equation is shown in Eq. (18).

(18) $$Kernel\;trick,k\left(x_{i},x_{j}\right)=x_{i}.x_{j}$$

**K-Nearest Neighbor (KNN):** KNN is an ML technique for supervised learning that is predicated on the idea that new data will be comparable to existing data. When adding new material, KNN makes sure to file it under the heading that is most comparable to the other available choices. Distance between nodes is calculated using the Manhattan equation in KNN. The Manhattan formula is displayed in Eq. (19).

(19) $$Manhattan\;distance:\sum_{i=1}^{k}\;\left|x_{i}-y_{i}\right|$$

**Random Forest (RF):** RF can be used for both classification and regression, as it is a supervised ML technique. To make a prediction, RF combines the outputs of many decision trees. This is why the term “ensemble model” is also used to describe RF. The results of various decision trees applied to multiple subsets of the dataset are compiled in this model. Taking their values and averaging them is done with the goal of improving the accuracy of forecasts based on the dataset. Using this method, a much larger number of trees can be evaluated with high precision.

**Naïve Bayes (NB):** It is possible to calculate the likelihood of a hypothesis by applying Bayes' method, often known as Bayes' Law or Rule. The results are determined by the NB using Eq. (20) to classify the data.

(20) $$P\left(A\;|\;B\right)=\frac{P\left(B\;|\;A\right)\;P\left(A\right)}{P\left(B\right)}$$

## 3 Experimental results

This section of the manuscript demonstrates the application of ML-based algorithms to the proposed study's data analysis. The first part of the paper shows that ML-based models can significantly improve upon the performance of a pre-trained CNN framework. The second step involves analyzing the experimental data using the LOA approach and feature selector methods like RFE, PCA, and LDA. Finally, the effectiveness of various classifiers is evaluated and compared. In order to evaluate the effectiveness of the proposed model, a variety of performance assessment matrices, such as F1-score (F1), accuracy (A), recall (R), and precision (P) have been applied. Table 4 provides a numerical representation of the performance evaluation matrices.

**Table 4:** Representation of the performance evaluation matrices.

Metrics	Description
Accuracy	The percentage of correct predictions will be shown. $A=\frac{TP+TN}{FP+TP+FN+TN}\times 100$
Precision	Described as a process of measuring a model's quality. $P=\frac{TP}{TP+FP}\times 100$
Recall	Model quantity assessment definition. $R=\frac{TP}{TP+FN}\times 100$
F1-score	Displays the model's credibility and accuracy. $F1=2\times \frac{R\times P}{R+P}\times 100$

### 3.1 Performance analysis without feature selection and lion optimization algorithm (LOA)

**Fig. 11:**
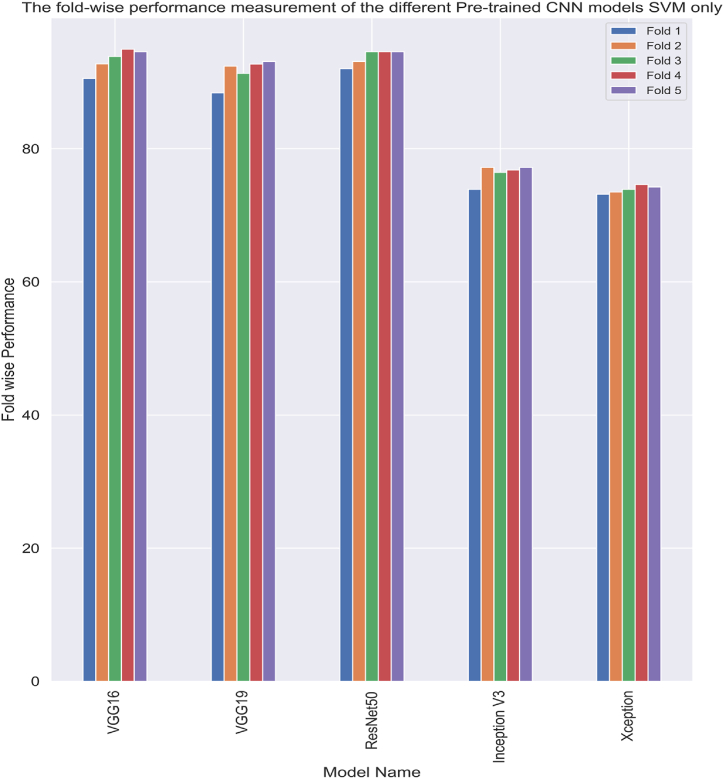
Analysis of the pre-trained CNN models' fold-wise results using SVM exclusively.

In this subsection of the article, the result analysis using the pre-trained CNN framework and the ML-based algorithms are shown. The proposed work's whole code base was executed on the Google Colab, which features a specialized GPU and 53 GB of RAM. The suggested model used the “pro” category of subscription of Colab. The study successfully extracted crucial information from the input image. Then the accumulated information was used in the evaluation of the ML-based models. To train and evaluate the suggested model, the dataset was divided into 80 % and 20 %. The proposed pre-trained CNN models with SVM method have been selected initially. To further improve performance, the study combines features to implement feature fusion using the Xception and InceptionV3 algorithms. A comparison between the SVM results with the suggested CNN models can be seen in Table 5. When compared to the other CNN models, ResNet50 performs the best with an accuracy of 98.55 %, followed by Xception (88.84 %), VGG16 (96.01 %), InceptionV3 (81.16 %), VGG19 (97.10 %). The proposed model employed 5-fold cross-validation strategies to determine the presence of overfitting problems, and the results are displayed in Fig. 11.

**Table 5:** The result analysis of the different pre-trained CNN models with SVM-only.

CNN Model	Accuracy	Precision	Recall	F1-score
VGG16	96.01	96.45	95.13	95.71
VGG19	97.10	97.30	96.56	96.90
**ResNet50**	**98.55**	**98.46**	**98.46**	**98.46**
Inception V3	81.16	83.17	76.89	78.11
Xception	88.84	89.09	85.57	76.79

Classic ML-based models (SVM, LR, DT, RF, XGB, NB, and KNC) were employed for the suggested study. Table 6 displays the whole output of the ML classifiers trained on the pre-trained CNN frameworks. The accuracy of 98.91 % is achieved by both of the pre-trained models, VGG16 and VGG19, combined with RF. In comparison to the other models, LR with ResNet50 produces the highest accuracy (99.28 %).

**Table 6:** Result analysis of different classifiers with pre-trained CNN models.

Classifiers	Accuracy			Precision			Recall			F1-Score		
	VGG16	VGG19	ResNet50	VGG16	VGG19	ResNet50	VGG16	VGG19	ResNet50	VGG16	VGG19	ResNet50
SVC	96.01	88.40	98.55	96.45	92.39	98.46	95.13	91.30	98.46	95.71	92.72	98.46
RF	98.91	98.91	96.74	99.14	98.94	96.81	98.57	98.76	96.27	98.57	98.84	96.52
DT	93.48	95.29	95.29	93.58	95.44	95.44	92.53	94.54	94.54	93.00	94.96	94.96
NB	86.59	88.41	89.13	85.92	87.61	88.28	87.89	89.54	90.13	86.26	88.07	88.79
XGB	98.55	98.55	98.55	98.86	98.86	98.86	98.10	98.10	98.10	98.45	98.45	98.45
KNC	97.46	97.46	96.38	97.39	97.39	96.73	97.22	97.22	95.61	97.30	97.30	96.11
LR	98.19	98.55	**99.28**	98.58	98.86	**99.42**	97.62	98.10	**99.05**	98.06	98.45	**99.23**

The comparison among the different pre-trained CNN techniques with the ML classifiers is displayed in Table 7. Compared to the other CNN models, the Extracted feature Vectors with ResNet50 + XGB combination achieve the best result of 99.28 % accuracy, which is shown in Table 7. Additionally, Table 8 displays the results of 5-fold cross-validation of extracted features through the pre-trained models and the ML classifiers.

**Table 7:** The comparison of the several techniques to analyze the performance achieved from different models.

Techniques	Accuracy	Precision	Recall	F1-score
Extracted Feature Vectors with VGG16+ RF	98.91	99.14	98.57	98.57
Extracted Feature Vectors with VGG19 + XGB	98.55	98.86	98.10	98.45
**Extracted Feature Vectors with ResNet50** **+** **XGB**	**99.28**	**99.42**	**99.05**	**99.23**
Extracted Feature Vectors + Xception + SVC	88.84	89.09	85.57	76.79

**Table 8:** The highest fold-wise result analysis of different pre-trained models with classifiers.

Techniques	Fold 1	Fold 2	Fold 3	Fold 4	Fold 5
Extracted Feature Vectors with VGG16+ RF	91.66	93.84	93.47	94.92	94.20
Extracted Feature Vectors with VGG19 + XGB	94.20	95.28	94.56	93.48	95.28
Extracted Feature Vectors with ResNet50 + XGB	92.02	95.28	94.56	94.91	95.28
Extracted Feature Vectors + Xception + SVC	73.18	73.50	73.91	74.63	74.27

**Fig. 12:**
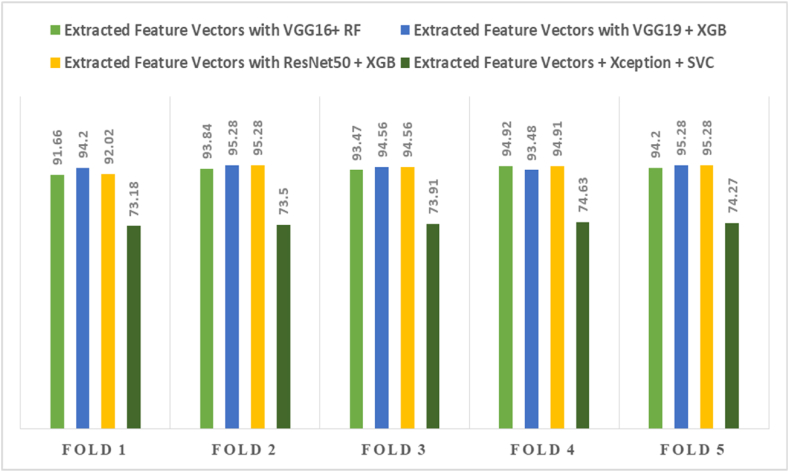
Comparison of accuracy of different CNN models.

**Fig. 13:**
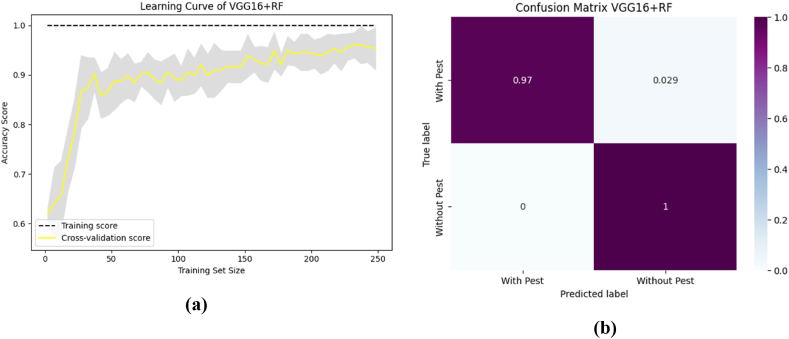
The highest performance measurement of VGG16 + RF with (a) Learning curve (b) Confusion Matrix.

**Fig. 14:**
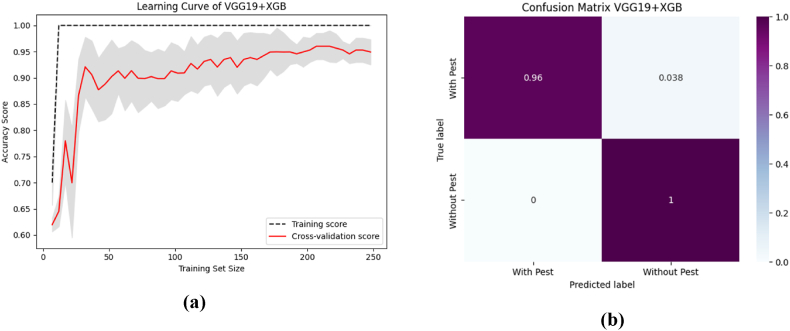
The highest performance analysis of VGG19 + XGB with (a) Learning curve (b) Confusion Matrix.

**Fig. 15:**
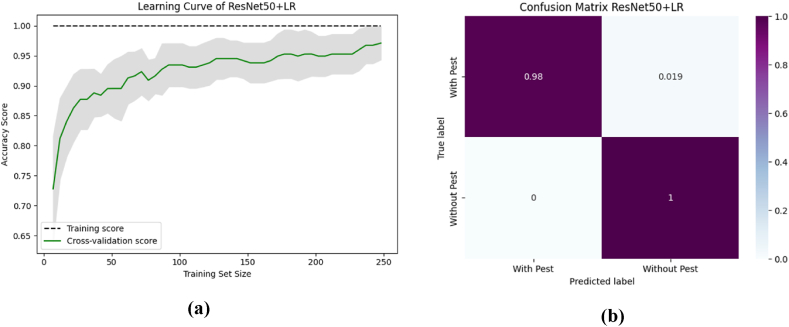
The performance analysis of ResNet50 + XGB with (a) Learning curve (b) Confusion matrix.

To aid comprehension, Fig. 12 shows the fold-wise performance evaluation matrices, and Fig. 13(a and b), Fig. 14 (a, b), and Fig. 15 (a, b) of the study provides a visual representation including training accuracy, learning curve, and confusion matrix, respectively.

### 3.2 Performance analysis with the lion optimization algorithm (LOA)

**Fig. 16:**
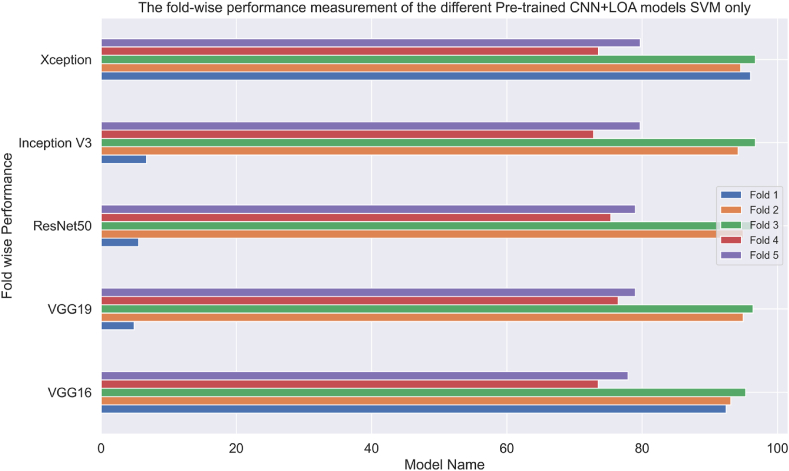
Analysis of the pre-trained CNN models' fold-wise results of CNN models with LOA and SVM only.

In this subsection, the research that has been proposed details the outcome analysis using the LOA method. The LOA approach was used after initially extracting features with the assistance of pre-trained CNN algorithms. After that, the suggested work analyzed the results. While considering the LOA, a set of parameters has been enumerated and tuned while working with the feature selection with the LOA. These are *population_size=500, nomad_ratio=0.2, num_of_prides=5, female_ratio=0.8, roaming_factor=0.2, mating_factor=0.3, mutation_factor=0.2, and immigration_factor=0.4*. We have applied the classifiers after the feature selection and after the parameter tuning. The overview of the data analysis for this investigation using LOA with CNN and SVC was provided in Table 9. In comparison to other models, the suggested method achieved the highest accuracy (99.92 %) when using ResNet50 + SVC + LOA. In contrast, the other models (VGG16 + SVC + LOA, VGG19 + SVC + LOA, Xception + SVC + LOA, and InceptionV3+SVC + LOA) only manages 98.19 %, 98.19 %, 89.86 %, and 81.52 % accuracy. Learning curves, 5-fold cross-validation, and a confusion matrix are all used to calculate the study's findings. Each of the pre-trained models can extract a different number of features from an input image, with VGG16, VGG19, ResNet50, InceptionV3, and Xception capable of extracting features, respectively. Table 9 displays the number of best features extracted by each of the pre-trained models; VGG16, VGG19, ResNet50, InceptionV3, and Xception respectively extracted 2057, 2013, 1007, 1013, and 1023 features. All the pre-trained CNN + SVC + LOA cross-validation data are displayed in Fig. 16.

**Table 9:** The overall performance of different pre-trained CNN models with SVM-only.

CNN Model	No. of best-selected features	Subset Accuracy	Accuracy	Precision	Recall	F1-score
VGG16	2057	98.18	98.19	97.95	98.15	98.05
VGG19	2013	98.55	98.19	98.15	97.94	98.04
**ResNet50**	**1007**	**99.92**	**98.81**	**98.93**	**98.72**	**98.83**
Inception V3	1013	82.24	81.52	83.29	76.64	78.25
Xception	1023	90.21	89.86	91.76	86.77	88.46

**Fig. 17:**
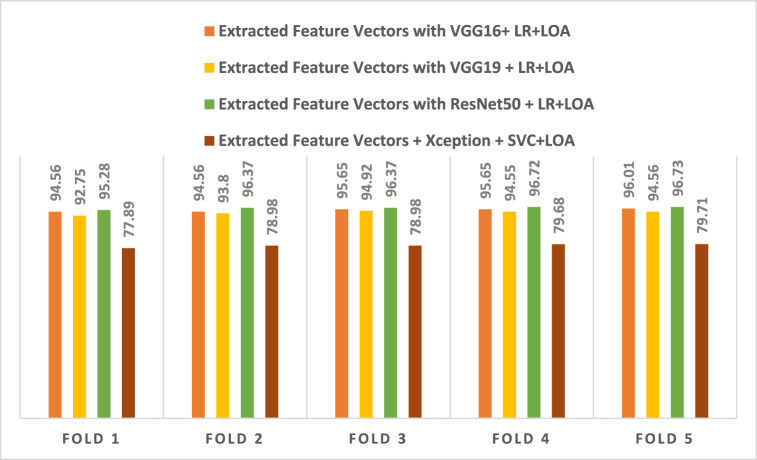
Comparison of accuracy of different CNN models with LOA.

In addition to that, Table 10 displays all of the outcomes achieved by the ML classifiers when used in conjunction with the pre-trained CNN architectures and LOA. Table 11 shows that the accuracy of LR quipped with VGG16 + LOA is 98.81 %, and also, the LR combined with VGG19 + LOA delivers an accuracy of 98.91 %. In comparison to the other models, SVC with ResNet50 + LOA produces the highest accuracy (99.81 %). Table 11 contrasts these results with a comparison of several ML algorithms applied to pre-trained CNN models. When compared to the other CNN models, the Extracted Feature Vectors with VGG19 + LR + LOA combination achieve the best result of 98.91 % accuracy. Additionally, the fold-wise performance of the different pre-trained models with the ML classifiers is illustrated in Table 12, and a visual representation of Table 12 is shown in Fig. 17.

**Table 10:** The overall performance of different classifiers with pre-trained CNN models and LOA.

Classifiers	Accuracy			Precision			Recall			F1-score		
	VGG16	VGG19	ResNet50	VGG16	VGG19	ResNet50	VGG16	VGG19	ResNet50	VGG16	VGG19	ResNet50
SVC	98.19	98.19	**98.81**	97.95	98.15	**98.93**	98.15	97.94	**98.72**	98.05	98.04	**98.83**
RF	97.46	98.55	97.83	97.18	98.65	97.48	97.37	98.23	97.48	97.27	98.43	97.87
DT	95.29	96.01	94.93	94.70	95.62	94.53	95.24	95.81	94.53	94.96	95.71	94.53
NB	89.86	92.75	92.75	88.79	91.69	91.69	91.16	93.45	93.45	89.45	92.37	92.37
XGB	97.83	97.46	98.19	97.48	97.18	97.79	97.87	97.37	98.36	97.67	97.27	98.06
KNC	97.83	97.46	96.74	97.66	97.36	97.27	97.66	97.16	95.75	97.66	97.26	96.43
LR	98.81	98.91	98.55	98.93	98.93	98.44	98.72	98.72	98.44	98.83	98.83	98.44

**Table 11:** Comparison among different pre-trained techniques with classifiers and LOA.

Techniques	Accuracy	Precision	Recall	F1-score
Extracted Feature Vectors with VGG16+ LR + LOA	98.81	98.93	98.72	98.83
**Extracted Feature Vectors with VGG19** **+** **LR** + **LOA**	**98.91**	**98.93**	**98.72**	**98.83**
Extracted Feature Vectors with ResNet50 + SVC + LOA	98.81	98.93	98.72	98.83
Extracted Feature Vectors + Xception + SVC + LOA	89.86	91.76	86.77	88.46

**Table 12:** The highest fold-wise performance of different pre-trained models with classifiers.

Techniques	Fold 1	Fold 2	Fold 3	Fold 4	Fold 5
Extracted Feature Vectors with VGG16+ LR + LOA	94.56	94.56	95.65	95.65	96.01
Extracted Feature Vectors with VGG19 + LR + LOA	92.75	93.80	94.92	94.55	94.56
Extracted Feature Vectors with ResNet50 + LR + LOA	95.28	96.37	96.37	96.72	96.73
Extracted Feature Vectors + Xception + SVC + LOA	77.89	78.98	78.98	79.68	79.71

**Fig. 18:**
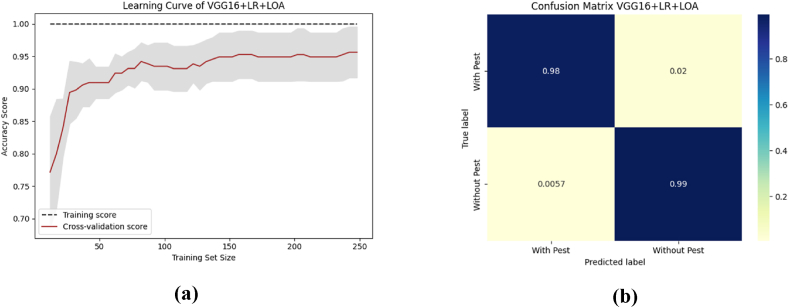
The highest performance measurement of VGG16 + LR + LOA with (a) Learning curve (b) Confusion matrix.

**Fig. 19:**
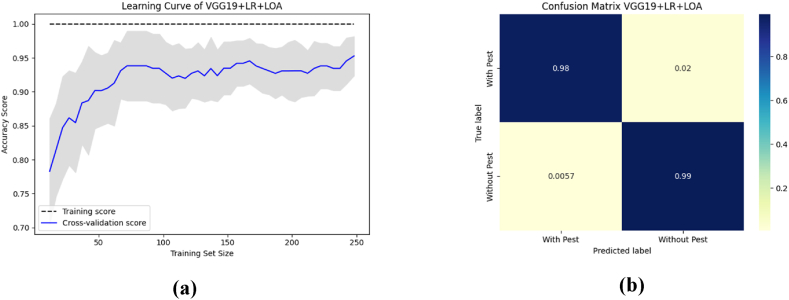
The highest performance measurement of VGG19 + LR + LOA with (a) Learning curve (b) Confusion matrix.

**Fig. 20:**
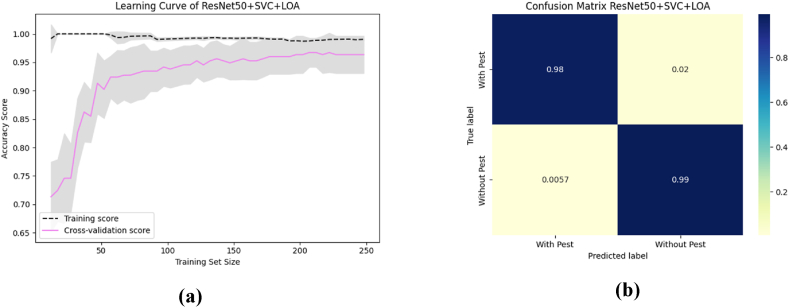
The performance measurement of ResNet50 + SVC + LOA with (a) Learning curve (b) Confusion matrix.

To aid comprehension, Fig. 18(a and b), Fig. 19 (a, b), and Fig. 20 (a, b) of the study provides a visual representation including accuracy, learning curve, and confusion matrix of Table 12.

Moreover, a comparison of acquired results with LOA and without LOA algorithm with ResNet50 and VGG19 of the proposed study is illustrated in Table 13. It is clearly shown in Table 13 that the result without LOA algorithm is better than the with LOA algorithm.

**Table 13:** Comparison of the performance of ResNet50 with and without LOA.

	Techniques	Accuracy	Precision	Recall	F1-score
Without LOA	Extracted Feature Vectors with ResNet50 + XGB	99.28	99.42	99.05	99.23
With LOA	Extracted Feature Vectors with VGG19 + LR + LOA	98.91	98.93	98.72	98.83

### 3.3 Performance analysis with the feature selection and the lion optimization algorithm (LOA)

**Fig. 21:**
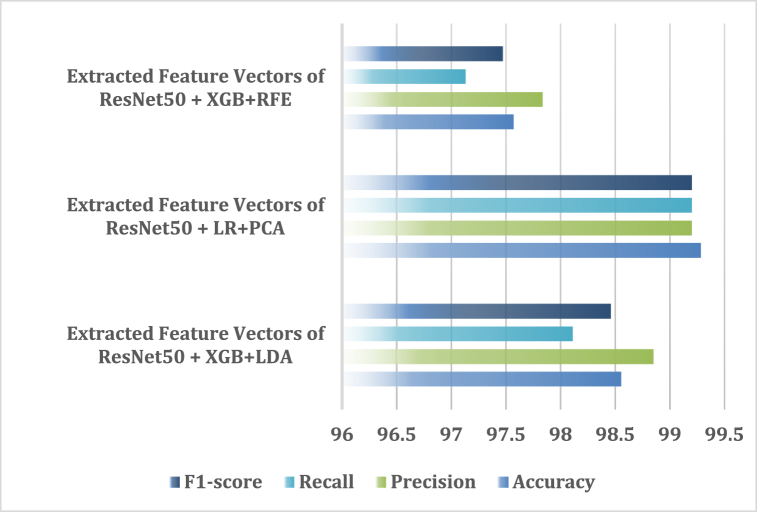
Comparison of accuracy of the ResNet50 model with different feature selectors and classifiers.

This section contains a discussion of the results of the suggested model's analysis using a number of different feature reduction algorithms, specifically LDA, PCA, and RFE feature selectors. This research data analysis is completed, and it employs a feature selector, ML models, and pre-trained CNN algorithms. Operations with the suggested model have been carried out using VGG16, VGG19 and ResNet50 features and the feature selector models initially. Data from experiments with several feature selector techniques is presented in Table 14. According to Table 14, the combined accuracy of the Extracted Feature Vectors of VGG16 + RF + LDA and Extracted Feature Vectors of ResNet50 + XGB + LDA method is 98.55 %. On the other hand, the accuracy of the Extracted Feature Vectors of ResNet50 + LR + PCA and Extracted Feature Vectors of ResNet50 + XGB + RFE are respectively 99.28 % and 97.57 %. Fig. 21 is a visual representation of a comparison between the Extracted Feature Vectors of ResNet50 with several ML models and feature selectors for identifying the insect accurately.

**Table 14:** The highest fold-wise performance of different pre-trained models with feature selectors and classifiers.

Feature Optimization Algorithms	Techniques	Accuracy	Precision	Recall	F1-score
LDA	**Extracted Feature Vectors of VGG16** **+** **RF**	**98.55**	**98.23**	**98.65**	**98.43**
	Extracted Feature Vectors of VGG19 + SVC	97.83	98.10	97.14	97.59
	**Extracted Feature Vectors of ResNet50** **+** **XGB**	**98.55**	**98.85**	**98.11**	**98.46**
PCA	Extracted Feature Vectors of VGG16 + SVC	98.91	99.15	98.54	98.83
	Extracted Feature Vectors of VGG19 + XGB	98.91	98.77	98.94	98.85
	**Extracted Feature Vectors of ResNet50** **+** **LR**	**99.28**	**99.20**	**99.20**	**99.20**
RFE	Extracted Feature Vectors of VGG16 + RF	97.45	97.25	97.65	97.44
	Extracted Feature Vectors of VGG19 + SVC	96.78	97.99	97.14	97.59
	**Extracted Feature Vectors of ResNet50** **+** **XGB**	**97.57**	**97.83**	**97.13**	**97.47**

## 4 Discussion

This section describes the comparison between the exiting works and the suggested architecture of identifying paddy insects, which is illustrated in Table 15. All the precursory work only used Deep Learning techniques for insect detection. Some of the previous studies [18,25], and [27] have achieved good accuracy of 98.28 %, 97.12 %, and 95.48 % among all of them. Additionally, some former papers [18,26,27], and [28] did not use the optimization techniques. The research [16,19] utilized the IP102 dataset for insect identification and classification. For detecting paddy insects, the paper [17] employed the paddy leaf images dataset, consisting of 400 pictures of diseased rice leaves, which is collected from a secondary source. Additionally, the work [18] validated the paddy insect classification model by applying it to the dataset obtained from intelligent insect forecasting lamps. To identify the paddy insect, paper [26] used good and infected paddy images of 27500 with 102 classes of insect. As for the feature selection techniques no existing work utilized this approach.

**Table 15:** The comparison among the existing works and the proposed architecture.

Ref	Use Algorithm/Model	Techniques	Feature Selection Algorithm	Optimization Methods	Accuracy
[16]	Transfer Learning, ResNet50, VGG16, and MobileNet	DL	–	Adaptive moment estimation (Adam) optimizer	84.39 %
[18]	Fully Convolutional Networks (FCNs), DenseNet	DL	–	–	98.28 %
[19]	Transfer Learning, Inception v3, Xception, VGG19, VGG16, and ResNet50	DL	–	Stochastic Gradient Descent (SGD) optimizer	82.5 %
[25]	VGG16, Inception V3, SqueezeNet, MobileNet, and NasNet	DL	–	Adaptive moment estimation (Adam) optimizer	97.12 %
[26]	Deep Transfer Learning, AlexNet	DL	–	–	89.33 %
[27]	Convolutional Neural Network	DL	–	–	95.48 %
[28]	Convolutional Neural Network, ZF Net, VGG16, and VGG19	DL	–	–	89.22 %
**Proposed Model**	**Inception v3, Xception, VGG19, VGG16, ResNet50, XGB, LR, DT, KNN, SVM, NB, and RF**	**DL** + **ML**	**LDA, PCA and RFE**	**Lion Optimization Algorithm (LOA)**	**99.28** **%**

On the other hand, the proposed work used ML and DL techniques with feature selector algorithms and optimization techniques to identify and classify paddy pests automatically. The collected dataset of 648 images for the present research has two categories one is paddy with pest (yes type), and the other one is paddy without pest (no type). Five pre-trained CNN-based algorithms (VGG16, VGG19, ResNet50, Inception V2, and Xception) were used in the present approach for feature extraction and interpretation after implementing image filtering and augmentation techniques to the dataset. After that, feature selection techniques such as PCA, RFE, and LDA will be implemented with the optimization algorithm LOA to get the best features from the extracted features of pre-trained frameworks. In the end, the best features are fed to the seven ML classic algorithms to monitor the identified pests and their classifications. The experimental results of the study indicated that the Extracted Feature Vectors of the ResNet50 + LR + PCA model achieved the maximum accuracy of 99.28 % among all the models.

Additionally, the combined accuracy of the Extracted Feature Vectors of VGG16 + RF + LDA and Extracted Feature Vectors of ResNet50 + XGB + LDA method is 98.55 %. Also, the accuracy of the Extracted Feature Vectors of ResNet50 + XGB + RFE is respectively 97.57 %. Lastly, the experimental results show that the current framework for identifying and classifying the paddy insect has given the best results through performance analysis, and the study also significantly advanced the state-of-the-art methods in this field.

## 5 Conclusion and future work

Certainly, paddy crops have significantly contributed to the world economy. The paddy farming industry is quickly becoming one of the most important economic sectors in Asia. This is primarily due to the high production rates and consistent profit margins that it offers. Destruction-causing insects, on the other hand, have a disproportionately negative effect on the usual growth rate of crops and the earnings that are anticipated from those crops. Hence, it is of utmost significance to possess a prompt and dependable technique for the identification of insects during rice harvesting. That is why the current study provides a solution for insect diagnosis by incorporating the mechanism of DL, ML and feature optimization model through optimizing the deep features of paddy insect images. The suggested solution will utilize five pre-trained CNN-based algorithms for the purpose of feature extraction and interpretation from the paddy images. Subsequently, the feature selection approaches, including PCA, RFE, and LDA, will be applied with the optimization algorithm LOA to obtain the most optimal features for the detection and categorization of paddy pests. Ultimately, a total of seven machine learning algorithms will be employed to accurately detect and classify pests that affect paddy crops. The suggested work shows all of the corresponding findings by employing the pre-trained algorithms with machine learning classifiers as well as the feature selection algorithms with the optimization method. The proposed framework indicated that the Extracted Feature Vectors of ResNet50 with LR and PCA have achieved the maximum accuracy of 99.28 % in identifying the paddy insects. Based on the experimental results, the proposed framework demonstrates precise and consistent identification of the paddy insect. Nevertheless, the present study will significantly influence the diagnosis of paddy insects when implemented in real-world scenarios.

However, there are some issues with the proposed model despite the fact that it achieved the best outcomes. At first, the idea is not actually implemented in the process of recognizing the paddy insects. Secondly, the LOA was employed merely for the purpose of doing an analysis of the information that was retrieved. When this method is applied to the deeper layers of a modified CNN, the resulting model will be suitable for incorporation into transportable Internet of Things (IoT) devices such as smartphones and other smart devices. Future research will hopefully identify answers to these issues, strengthening the proposed system for practical application. Real-time datasets and other DL algorithms will be used in the future for paddy insect detection. In addition, ML and IoT methods will be used in the future to solve the problem of identifying destructive paddy diseases and forecasting crop yields. Lastly, object detection and encountering multi-object overlap tasks will be addressed and solved in the future.

## Funding statement

The authors are thankful to the Deanship of Graduate Studies and Scientific Research at 10.13039/501100005911Najran University for funding this work under the Elite Funding Program grant code (NU/EP/SERC/13/776).

## Data availability

All the data is available within the document.

## CRediT authorship contribution statement

**M.A. Elmagzoub:** Writing – original draft, Methodology, Investigation, Formal analysis, Data curation, Conceptualization. **Wahidur Rahman:** Writing – original draft, Methodology, Investigation, Formal analysis, Data curation, Conceptualization. **Kaniz Roksana:** Writing – original draft, Methodology, Investigation, Formal analysis, Data curation, Conceptualization. **Md. Tarequl Islam:** Writing – original draft, Methodology, Investigation, Data curation, Conceptualization. **A.H.M. Saifullah Sadi:** Writing – review & editing, Methodology, Investigation, Formal analysis, Data curation, Conceptualization. **Mohammad Motiur Rahman:** Writing – review & editing, Resources, Project administration, Methodology, Investigation. **Adel Rajab:** Writing – review & editing, Resources, Project administration, Methodology, Investigation, Conceptualization. **Khairan Rajab:** Writing – review & editing, Resources, Project administration, Methodology, Investigation. **Asadullah Shaikh:** Writing – review & editing, Supervision, Resources, Project administration, Methodology.

## Declaration of competing interest

The authors declare that they have no known competing financial interests or personal relationships that could have appeared to influence the work reported in this paper.
